# Medical Etiquette

**Published:** 1885-10

**Authors:** C. C. Francis

**Affiliations:** Cleburne, Texas


### Medical ★ Journal,

PUBLISHED MONTHLY AT

AUSTIN, TEXAS.

Vol. 1.] OCTOBER, 1885. [No. 4.

*"Scribimus indocti, doctique!"*

### Original Articles.

☞ CONTRIBUTED EXCLUSIVELY TO THIS JOURNAL. ☜

*The Articles in this Department are accepted, and published with the understanding that we are not responsible for, nor do we indorse the views and opinions of the writers, by so doing.*

### MEDICAL ETIQUETTE.

*By C. C. Francis, M. D., Cleburne, Texas.*

Read before the Johnson County Medical Association. For Daniel's Medical Journal.

IN presenting to the Johnson County Medical Association an essay upon certain rules and regulations governing the relations that exist between medical men, and the professional duties that are due from one member to another under all circumstances, which is a question of difficult solution, it is not my intention to submit a code of laws applicable to the government of the profession, but merely to offer a few thoughts upon some of the rules that may be conducive to a more perfect understanding and good will that should at all times pervade the daily intercourse of the busy practitioner with his medical brethren.

After many years of observation and experience I have serious doubts as to the successful establishment and adaptation of any code of medical ethics that will place the brethren in the profession upon a perfectly satisfactory and harmonious basis.

There exists no standard work on the subject that I am aware of—the only rules are those emanating from the American Medical Association, and such as are published through the medical press. The rules established and formulated by the American Medical Association must of necessity take a wide range; consequently they cannot be framed so as to meet the requirements suitable for all localities. I hold that these laws are established upon correct principles, and are wholesome as general laws govering the medical profession, that they should be adopted and strictly adhered to by our County Medical Societies; in addition to which each county association should engraft thereon such other rules and regulations as would have a tendency to develop a more perfect understanding as to what constitutes true manhood and professional courtesy; that when they are established and adopted, they should be printed, and each member furnished with a copy.

Much of the ill feeling existing among the members of the profession is the result of a misconception of what constitutes medical etiquette. Correct and incorrect opinions are entertained and acted upon—the one taking a correct and the other, honestly, an incorrect view. Under these circumstances it would be difficult to come to a satisfactory understanding. One presumed professional discourtesy from slight causes may lead to others, and utlimately result in an estrangement between personal and professional friends that would never have existed, had a thorough and complete code of laws been agreed upon and understood.

Much of the misunderstanding that is engendered among the medical profession may be attributed, in a great measure, to the want of a knowledge on the part of the people in regard to the relation one medical man sustains to another, as well as the relation he sustains to the patient, and the community generally.

The physician has rights, and is entitled to due respect in the line of his profession; likewise the patron and patient have certain rights which should be conceded. These several rights should be defined and incorporated in the laws regulating the profession; the same should be published, so as to be read and understood by the people as well as the profession. When we have once instructed them as to their rights, and the respect they are due the profession, we will, in a great measure, have throttled one of the prime causes underlying nine-tenths of all the hatred and bickerings that have ever existed among medical men.

Here it is that the unprincipled charlatan, either by direct thrusts or the more potent agent, insinuation, stabs his professional brother in the "dark." He makes it a point never to agree when called in consultation. Should the course of treatment be correct, the mode of administration must be changed; pills must be substituted for powders, or vice versa, powders for pills. If aconite is being administered, gelsemium or veratrum would meet the indications more satisfactorily; syrup of tolu is wonderfully healing and stimulating to bronchial mucous membranes, and will greatly add to the efficiency of the expectorant prescribed; a runner must be sent post haste to the drug store to make the impression more profound. If the patient recovers, his timely assistance and scientific knowledge saved him. If he dies, it was the result of his valuable aid not being demanded sooner; he could see just where his professional brother made the fatal mistake, which could have been obviated at the proper time.

Another ingenious and oft practiced method of those who hope to rise upon the downfall of others, is very frequently resorted to by this class of men. Well, Dr. A., what do you think of Dr. B., who has just settled in our town? With a knowing look and half sarcastic smile, he replies: W-e-l-l, I d-o-n't k-n-o-w. I should think he is rather young yet, to be skillful. He'll do very well for simple diseases, such as chills and fever. I don't think it would be safe to risk him in a bad case; he may make a good doctor when he gets age and experience. Well, there is old Dr. C., who has settled in our midst, what sort of a doctor is he? Oh, well, you can see for yourself —probably he's been a good doctor in his day—he's a regular old fogy—hasn't kept up with the profession. I'd be afraid to risk him. These and other similar remarks, together with direct and indirect insinuations resorted to by this class of medical men, are readily caught on to by the ignorant masses, and especially by some knowing old woman, of which nearly every neighborhood is the happy possessor. They "roll it as a sweet morsel under their tongues;" no opportunity is lost, not only to perpetuate what Dr. A. has said about Drs. B. and C., but exagerated statements are made. The bold, embrazoned, unprincipled Dr. A. stands before the community as a man of great sagacity and wonderful medical attainments, while the quiet, honest, conscientious, worthy physician, is sacrificed upon the altar of justice, at the hands of an ignorant and misguided community.

But thanks for the old adage, "Truth is mighty and will prevail," "murder will out." The misguided will live to see their errors, the unprincipled man his own downfall, and the meritorious will occupy a position worthy of his honesty and attainments.

Medical etiquette has for its object the establishment of a system of laws by which medical men may know and understand the duties they owe to each other, and when strictly adhered to, as they should be, could not do otherwise than produce harmony, and good feeling, binding them happily together in the prosecution of a noble and glorious work.

Whenever we loose sight of an honest, upright and straightforward course, either for pecuniary gain or to establish a reputation for skill and ability, we transcend the rules of propriety, and subject ourselves to a just censure from all honorable men. The feeling of an ambition to excel is commendable, but the successful and thoroughly educated medical man should not boast of his attainments; let him be content to know and feel that he is able to cope with the best minds in the profession, ever ready to extend aid to those in search of knowledge.

Our profession, like all others, is made up of men whose intellect, learning and wisdom are reckoned by the extremes. The weak, as well as the strong, should at all times stand upon the watch tower, ever ready to cement the ties that should bind us together as a noble brotherhood, standing between life and death.

There is no calling more honorable, no profession more laborious, and none that requires as much patience and good will amongst its members, as that of the medical profession.

We should never let such trifles as a medical man, moving into our town, or some adjacent neighborhood,(although it may limit our practice) influence us in our friendly feelings toward him. If he merits a practice, let him have it. If not, the people must be the judge, and not the professional man's business to censure.

Quacks and charlatans should receive no mercy or recognition at the hands of the profession—likewise Homœopathy. The Allopath (so called) and Homœopathy are as distinct as day and night, consequently it is useless to attempt to make *something* recognize *nothing* as a science worthy of the confidence of an intelligent people.

On the other hand we should be true to each other, and especially ever ready and willing to aid and sustain the honorable young men of the profession.

If we expect them to grow up as respeoted members, they must be dealt with justly, encouraged and sustained by those who have grown old and experienced; ever remembering that we too were once young, and had to commence at the foot of the ladder.

I fear, of late, that we have lost much of that true and exalted impulse that encouraged and fostered the profession in the search of knowledge during the days of its infancy.

That the profession, at a very remote age in its history, were closely allied, and harmoniously linked together, we have only to refer to writings of that venerable teacher, and one of the fathers of medicine, Hippocrates; here we will find that no narrow-minded or exclusive selfishness controlled our ancient brethren; but a line of generous conduct and morality was enjoined on all alike.

In order to refresh our minds I will close this short and imperfect essay by quoting the Hippocratic oath :

[Oath omitted for want of space.—Ed.]

